# Short-term clinical performance of CAD/CAM hybrid composite and conventional indirect composite onlays in posterior teeth: A retrospective cohort study

**DOI:** 10.4317/jced.64450

**Published:** 2026-09-15

**Authors:** Van Nhat Thang Le, Tran Bao Nguyen, Minh Huy Dang

**Affiliations:** 1 Faculty of Odonto-Stomatology, University of Medicine and Pharmacy, Hue University, Hue City, Vietnam

**Keywords:** CAD/CAM, hybrid composite, indirect composite, onlay, posterior teeth.

## Abstract

**Background:**

CAD/CAM hybrid composite onlays are increasingly used for posterior indirect restorations. Although related clinical studies have evaluated CAD/CAM hybrid composite, direct comparisons with conventional indirect composite onlays remain limited. This retrospective cohort study compared the short-term clinical performance of two posterior onlay workflows: CAD/CAM hybrid composite milling and conventional indirect composite layering.

**Materials and Methods:**

Posterior onlays placed between May 2022 and March 2024 were reviewed in a single-centre clinical setting. A total of 66 restorations in 60 patients were analysed: 33 conventional indirect composite onlays and 33 CAD/CAM hybrid composite onlays. The comparison was interpreted as a workflow comparison because material type and fabrication pathway differed simultaneously. Baseline comparability between groups was evaluated using standardised mean differences for age, gender, tooth position, cavity class, cusp involvement and pulpal status. The principal outcome was 6-month marginal fit assessed using modified USPHS criteria. Colour/gloss rating, proximal contact integrity and restoration integrity were secondary outcomes.

**Results:**

At 6 months, CAD/CAM hybrid composite onlays showed higher unadjusted proportions of Alpha ratings for marginal fit than conventional indirect composite onlays (84.8% vs. 57.6%; p=0.015). More favourable unadjusted ratings were also observed for colour/gloss and proximal contact integrity. However, baseline marginal fit already favoured the CAD/CAM group, and after adjustment for baseline marginal fit and baseline covariates, the association with 6-month Alpha marginal fit was no longer statistically significant. Restoration integrity was numerically higher in the CAD/CAM group, but the difference was not significant. One secondary caries case occurred in the conventional group, and no debonding was recorded.

**Conclusions:**

Both workflows achieved clinically acceptable short-term outcomes. CAD/CAM hybrid composite onlays showed more favourable unadjusted 6-month ratings, but the adjusted association with the principal outcome, marginal fit, was not statistically significant. Baseline imbalance, residual confounding, workflow-related differences, small sample size and short follow-up preclude conclusions of superiority. Prospective controlled studies with longer follow-up are needed.

## Introduction

Minimally invasive posterior indirect restorations, including inlays and onlays, have become an important conservative alternative to full-coverage crowns for teeth with extensive coronal tissue loss [1,2]. By preserving sound tooth structure and relying on adhesive bonding, these restorations allow clinicians to restore function, morphology and occlusal stability while avoiding unnecessary removal of enamel and dentin. Nevertheless, their clinical performance is multifactorial and depends not only on the amount and quality of remaining tooth structure, but also on cavity design, adhesive procedures, restorative material, fabrication workflow, occlusal loading and operator-related factors.

Resin-based indirect restorations remain a practical option for posterior teeth because of reparability, conservative preparation requirements and compatibility with adhesive techniques. Conventional indirect composite onlays are typically fabricated by incremental layering and light-curing on a working cast. Although this approach is accessible and widely applicable, it may be affected by manual fabrication variability and polymerisation-related limitations. In contrast, CAD/CAM hybrid composite onlays are milled from industrially polymerised blocks. High-temperature and high-pressure polymerisation has been associated with improved monomer conversion, lower residual monomer content and reduced polymerisation shrinkage [3,4], while some CAD/CAM hybrid materials have been reported to exhibit mechanical characteristics closer to dentin and enamel than ceramic blocks [5,6].

Despite the growing adoption of CAD/CAM resin-based restorative materials, direct clinical evidence comparing CAD/CAM hybrid composite onlays with conventional indirect composite onlays remains limited. Related studies have reported acceptable short- to medium-term outcomes for CAD/CAM resin-based or hybrid composite restorations [7-9], and recent clinical studies have compared related indirect restorative strategies, including CAD/CAM composite versus lithium disilicate and direct versus indirect onlays [10,11]. Thus, the contribution of the present study is focused rather than wholly novel: It examines two distinct posterior onlay workflows under the same routine-care setting. Few investigations have directly compared CAD/CAM hybrid composite onlays with conventional indirect composite onlays, in which both material and fabrication pathway differ. This focused gap is clinically relevant because treatment selection in everyday practice is often shaped by patient preference, cost, material availability, laboratory workflow and clinician experience. Observational data from routine care may therefore provide useful complementary evidence by reflecting how these restorative approaches perform in real-world clinical settings.

However, retrospective studies based on routinely collected clinical data require careful interpretation because treatment allocation is not randomised and confounding may influence the observed associations. Residual confounding from unmeasured clinical and laboratory factors cannot be fully eliminated [12]. Therefore, the present study aimed to compare the short-term clinical ratings of CAD/CAM hybrid composite and conventional indirect composite onlays in posterior teeth placed in routine practice. Because the treatments differed in both material composition and fabrication pathway, the study was designed as a workflow comparison rather than a material-only comparison. Marginal fit at 6 months was defined as the principal outcome, while colour/gloss rating, proximal contact integrity and restoration integrity were evaluated as secondary outcomes [13].

The null hypothesis was that there would be no statistically significant difference in 6-month clinical performance, specifically marginal fit, between CAD/CAM hybrid composite onlays and conventional indirect composite onlays in posterior teeth.

## Materials and Methods

*Study design and reporting*

This single-centre retrospective cohort study compared CAD/CAM hybrid composite and conventional indirect composite onlays placed in posterior teeth at the dental clinic of Hue University of Medicine and Pharmacy Hospital, Vietnam. Routinely collected clinical data were retrieved from electronic and paper-based records. Six-month outcomes were obtained from documented recall visits or, when required, from a standardised clinical re-examination. The study was reported in accordance with the STROBE statement [12].

*Study period, data sources, and ethical considerations*

Eligible restorations were placed between May 2022 and March 2024. Data extraction and 6-month re-examinations were performed between April and September 2024. Data sources included clinical records, structured evaluation forms, clinical photographs, periapical radiographs, and direct 6-month clinical assessments. The study protocol was approved by the Ethics Committee of Hue University of Medicine and Pharmacy, Hue University, Hue City, Vietnam (Approval No. H2022/250) and complied with the Declaration of Helsinki. The requirement for informed consent was waived for the retrospective chart-review component. Written informed consent was obtained from all participants who underwent 6-month re-examination. For participants younger than 18 years, written informed consent was obtained from a parent or legal guardian, with participant assent obtained when applicable.

*Participants and eligibility criteria*

The restoration was the primary unit of analysis. Posterior onlay restorations placed in patients aged 15-60 years, excluding third molars, were assessed against the following criteria. Inclusion criteria were as follows: Documented clinical indication for an onlay restoration [14]; fabrication from either Tetric N-Ceram (Ivoclar Vivadent, Schaan, Liechtenstein) or Shofu HC (Shofu Inc., Kyoto, Japan); complete baseline, 1-month and 3-month clinical evaluation data; available 6-month follow-up.

Exclusion criteria were as follows: Active periapical or furcation pathology; severe parafunction without occlusal protection; six-month outcome data not obtainable.

*Study groups*

Restorations were classified into two groups according to the material and fabrication workflow recorded in the clinical notes. The conventional indirect composite group consisted of Tetric N-Ceram onlays fabricated by incremental layering and light-curing on a working cast. The CAD/CAM hybrid composite group consisted of Shofu HC onlays milled from industrially polymerised blocks. Material selection was not randomised and was based on clinical indication, material availability, clinician judgement, and patient preference. Because material composition and fabrication pathway were inseparable in the recorded treatments, the exposure of interest was the complete restorative workflow. Accordingly, observed associations cannot be ascribed to material type alone.

*Restorative procedures*

All restorations were placed using a standardised adhesive protocol. Cusps thinner than 2 mm, or thinner than 3 mm in endodontically treated teeth, were reduced. Marginal ridges below 1.5 mm were also reduced. Preparations were designed to provide at least 1.5 mm of restorative material at the central fissure and 2.0 mm at reduced cusp areas. Immediate dentin sealing was performed using 37% phosphoric acid followed by FL-Bond II [15].

For both groups, impressions were made using a two-step putty-wash technique, poured in dental stone, and processed on laboratory working casts. Conventional indirect composite onlays were fabricated by incremental layering and light-curing of Tetric N-Ceram on the working cast. For CAD/CAM hybrid composite onlays, the stone casts were scanned in the laboratory, and the restorations were milled from Shofu HC blocks. Cementation was performed under rubber-dam isolation in all cases. Selective enamel etching and FL-Bond II application on dentin were followed by luting with pre-warmed composite cement. Each surface was light-cured for 20 s, followed by an additional 20 s of curing under glycerin gel. The restorations were then adjusted, finished, and polished.

*Outcome assessment*

Clinical outcomes were assessed using modified USPHS criteria [13,16] at baseline, 1 month, 3 months, and 6 months. The assessed criteria were marginal fit, occlusal fit, proximal contact, anatomic form, colour/gloss, marginal discolouration, restoration integrity, and secondary caries. Alpha indicated an ideal or clinically excellent rating, Bravo indicated a clinically acceptable rating, and Charlie indicated an unacceptable rating.

The principal outcome was Alpha-rated marginal fit at 6 months. Secondary outcomes were colour/gloss rating, proximal contact integrity, and restoration integrity. Other criteria and time points were considered exploratory. Data were extracted independently by two examiners, with disagreements resolved by consensus. The 6-month re-examination was performed by two calibrated examiners, with kappa values greater than 0.80, who were blinded to material allocation. Complete masking may not have been possible because of visible material- or workflow-related differences. Because no Charlie ratings were observed, outcomes were dichotomised as Alpha versus non-Alpha.

*Statistical analysis*

No a priori sample size or power calculation was performed because of the retrospective design, in which the analysis was based on a fixed set of eligible restorations; hence, the study was exploratory rather than powered to test a definitive superiority hypothesis. Categorical variables are presented as counts and percentages. Continuous variables are presented as means and standard deviations. Covariate balance was assessed using standardised mean differences rather than significance testing.

Alpha ratings were compared between groups at each time point using chi-square or Fisher’s exact tests, as appropriate. Changes over time within each group were analysed using Cochran’s Q tests. The primary analysis compared Alpha-rated marginal fit at 6 months between the two groups. A baseline-adjusted logistic regression model was also fitted for the principal outcome. The dependent variable was 6-month Alpha marginal fit, and the main independent variable was restorative workflow. The model adjusted for baseline marginal fit and the prespecified baseline covariates (age, gender, tooth position, Black cavity class, cusp involvement, and pulpal status). Because baseline differences in marginal fit and occlusal fit were present between groups, 6-month findings were interpreted in relation to baseline ratings. Given the limited sample size and number of non-Alpha outcomes, the adjusted analysis was exploratory; odds ratios and 95% confidence intervals were interpreted primarily for direction and precision rather than as definitive evidence. Secondary and exploratory outcomes were not adjusted for multiple comparisons and should be interpreted as hypothesis-generating. Given the limited sample size, the adjusted estimates were imprecise, as reflected in wide confidence intervals.

Intra-patient correlation was not modelled in the main analysis because only six patients contributed more than one restoration. A sensitivity analysis randomly retained one restoration per patient to examine whether this potential non-independence altered the direction of the primary unadjusted comparison. This sensitivity analysis does not fully replace a formal patient-level clustered model, and residual non-independence may persist. All tests were two-sided, with α=0.05. Analyses were performed using SPSS version 20.0.

## Results

*Study sample and baseline characteristics*

A total of 66 restorations in 60 patients were analysed, with 33 in each group: 33 conventional indirect composite onlays and 33 CAD/CAM hybrid composite onlays.

The mean age of the patients was 21.67 ± 5.22 years, with an age range of 15-47 years. Most patients were female, accounting for 78.3% of the sample, and 93.3% were younger than 30 years. All standardised mean differences were below 0.10, indicating adequate balance for the baseline covariates between the two groups (Table 1).

However, balance in baseline covariates did not completely translate into balance in baseline clinical ratings. Alpha ratings for marginal fit and occlusal fit were more frequent in the CAD/CAM hybrid composite group at baseline. Therefore, follow-up findings for these outcomes were interpreted in relation to the baseline ratings.

*Principal outcome: Marginal fit at 6 months*

At 6 months, Alpha ratings for marginal fit were more common in the CAD/CAM hybrid composite group than in the conventional indirect composite group (84.8% vs. 57.6%; *p*=0.015) (Table 2).

This difference was attenuated after adjustment for baseline marginal fit and the baseline covariates. In the baseline-adjusted logistic regression model, the association between restorative workflow and 6-month Alpha marginal fit was no longer statistically significant (adjusted OR=1.33; 95% CI: 0.21-8.33; *p*=0.761). The adjusted estimate was imprecise, and the unadjusted difference should therefore not be interpreted as evidence of clinical superiority.

The one-restoration-per-patient sensitivity analysis showed the same direction of the unadjusted association. However, because it was not a formal clustered analysis, it should be interpreted only as a robustness check.

A difference in marginal fit was already present immediately after cementation. At baseline, Alpha ratings were observed in 97.0% of CAD/CAM hybrid composite onlays and 75.8% of conventional indirect composite onlays (*p*=0.028). From baseline to 6 months, the proportion of Alpha ratings decreased by 18.2 percentage points in the conventional group and by 12.2 percentage points in the CAD/CAM hybrid composite group.

Thus, although CAD/CAM hybrid composite onlays showed a higher proportion of Alpha-rated marginal fit at 6 months, part of this unadjusted difference was already present at baseline. No conclusion can therefore be drawn that the CAD/CAM workflow produced better longitudinal marginal-fit performance.

*Secondary outcomes at 6 months*

At 6 months, the CAD/CAM hybrid composite group also showed higher proportions of Alpha ratings for the secondary outcomes (Table 2).

For colour/gloss rating, Alpha ratings were recorded in 75.8% of CAD/CAM hybrid composite onlays and 39.4% of conventional indirect composite onlays (*p*=0.003). At baseline, the proportions were closer, although the difference still favoured the CAD/CAM hybrid composite group (93.9% vs. 78.8%; *p*=0.144).

For proximal contact integrity, Alpha ratings at 6 months were observed in 81.3% of restorations in the CAD/CAM hybrid composite group and 57.6% in the conventional indirect composite group (*p*=0.037). Baseline proportions were not significantly different between groups (90.9% vs. 75.8%; *p*=0.105).

For restoration integrity, Alpha ratings were numerically higher in the CAD/CAM hybrid composite group than in the conventional indirect composite group, but the difference was not statistically significant (97.0% vs. 84.8%; *p*=0.195). One case of secondary caries was recorded in the conventional indirect composite group, whereas none occurred in the CAD/CAM hybrid composite group. No debonding was observed in either group.

*Exploratory outcomes and changes over time*

Exploratory comparisons are shown in Table 2. Occlusal fit differed between groups at baseline, with a higher proportion of Alpha ratings in the CAD/CAM hybrid composite group (90.9% vs. 60.6%; *p*=0.004). Because this difference was already present at baseline, follow-up comparisons for occlusal fit should not be interpreted as evidence of a purely longitudinal difference between the two restorative approaches.

Within-group changes over time are presented in Table 3. In the conventional indirect composite group, significant declines in Alpha ratings were observed for colour/gloss (*p*=0.001), anatomic form (*p*=0.024), marginal fit (*p*=0.041), proximal contact (*p*=0.047), marginal discolouration (*p*=0.049), and restoration integrity (*p*=0.030). In the CAD/CAM hybrid composite group, a significant decline was observed only for anatomic form (*p*=0.046).

## Discussion

In this retrospective cohort study of posterior indirect onlays placed in routine clinical practice, CAD/CAM hybrid composite onlays showed more favourable 6-month clinical ratings than conventional indirect composite onlays in unadjusted comparisons. However, the principal outcome, marginal fit, was not significantly associated with restorative workflow after adjustment for baseline marginal fit and measured baseline covariates. The study therefore does not support claims of clinical superiority of one workflow over the other.

These findings should be interpreted with caution. For the principal outcome, marginal fit already favoured the CAD/CAM hybrid composite group at baseline (97.0% vs. 75.8% Alpha; *p*=0.028). After adjustment for baseline marginal fit and the baseline covariates, the association between restorative workflow and 6-month Alpha marginal fit was no longer statistically significant (adjusted OR=1.33; 95% CI: 0.21-8.33; *p*=0.761). The wide confidence interval indicates substantial uncertainty. More favourable initial adaptation, differences in fabrication workflow, and unmeasured confounding are all plausible explanations for the unadjusted difference; the present study cannot distinguish their relative contributions.

Marginal adaptation is a clinically relevant criterion for posterior indirect restorations because inadequate marginal integrity may contribute to plaque accumulation, marginal discolouration, secondary caries and reduced restoration longevity. In the present study, CAD/CAM hybrid composite onlays had a higher proportion of Alpha ratings for marginal fit at 6 months in the unadjusted comparison. This observation overlaps with reports of acceptable short- to medium-term performance for CAD/CAM resin-based, hybrid composite, direct, and indirect onlay restorations [7-11]. Accordingly, the novelty of the present study is moderate and focused on the routine-care comparison of two distinct workflows, not on establishing a new material-specific effect. Direct comparison with previous studies should remain cautious because of differences in study design, restorative materials, cavity configurations, clinical protocols, evaluation criteria and follow-up duration.

The difference in colour/gloss rating was more pronounced at 6 months. The conventional indirect composite group showed a greater reduction in Alpha ratings over time, whereas the CAD/CAM hybrid composite group maintained more favourable ratings. This observation is consistent with evidence that resin composites may undergo colour changes after exposure to water and staining beverages [17,18]. Industrially polymerised CAD/CAM composite blocks may provide more homogeneous polymerisation, higher monomer conversion and lower residual monomer content than conventionally light-cured resin composites [3,4]. These material characteristics may partly explain the more favourable colour/gloss ratings observed in the CAD/CAM hybrid composite group. Nevertheless, the present study did not include instrumental colour measurement, surface roughness assessment or water sorption analysis; therefore, this explanation remains hypothetical.

Proximal contact integrity was also more favourable in the CAD/CAM hybrid composite group at 6 months. This difference may be related to greater standardisation of the CAD/CAM fabrication process and more consistent reproduction of restoration contour. However, proximal contact is influenced by several clinical and technical factors, including impression accuracy, working cast quality, restoration design, milling or laboratory procedures, cementation pressure, occlusal adjustment and physiological tooth movement. Because these factors were not fully controlled in this retrospective cohort study, the observed difference should be interpreted as an association rather than a direct effect of restorative material.

Several workflow-related factors may have contributed to the observed differences. Industrially polymerised CAD/CAM hybrid composite blocks may improve consistency and structural homogeneity compared with manually fabricated restorations [3-6]. Favourable mechanical and optical properties have also been reported for these materials [19-22]. However, the two groups differed not only in material composition but also in fabrication route and potentially laboratory handling. The relative contribution of these factors cannot be separated in the present retrospective data set. Therefore, the findings should be interpreted as associations with complete restorative workflows, rather than as material-only effects.

The clinical relevance of the observed differences should be interpreted in relation to the outcome scale. Because no Charlie ratings were recorded at any time point, modified USPHS outcomes were dichotomised as Alpha versus non-Alpha. The observed differences therefore mainly distinguished clinically excellent from clinically acceptable outcomes, rather than separating successful restorations from overt failures.

From a practical perspective, the two workflows differ in cost and infrastructure. CAD/CAM fabrication requires investment in scanning, design and milling, which may not be feasible in many middle-income settings, whereas conventional indirect composite onlays can be produced using widely available laboratory procedures and remain a more accessible option in Vietnam and similar contexts. The absence of Charlie ratings and debonding in both groups at 6 months suggests that clinically acceptable short-term outcomes can be achieved without CAD/CAM technology. These practical considerations should inform workflow selection for individual patients and clinical settings.

Clinically, both workflows appear to be reasonable short-term options for posterior indirect restorations in routine practice. However, the present findings do not support replacing conventional indirect composite onlays with CAD/CAM hybrid composite onlays or favouring one material on the basis of the observed unadjusted differences. Workflow selection should remain individualised according to remaining tooth structure, cavity design, pulpal status, occlusal load, adhesive protocol, available infrastructure, cost and patient preference.

This study has several strengths. First, the study was based on restorations placed in routine clinical practice, providing data from an everyday clinical setting. Second, the two comparison groups were comparable for the measured baseline covariates, with all standardised mean differences below 0.10. Third, the restorative procedures followed a standardised adhesive protocol, including immediate dentin sealing and adhesive cementation [15]. Fourth, clinical outcomes were assessed using modified United States Public Health Service criteria [13], and the 6-month assessment was performed by calibrated examiners.

Several limitations should also be acknowledged. First, treatment was not randomised and the workflows differed simultaneously in material composition and fabrication pathway. Therefore, residual confounding from unmeasured factors-including oral hygiene, socioeconomic status, operator experience, perceived case difficulty and laboratory variability-cannot be excluded, and baseline imbalance in marginal fit further limits causal interpretation of the principal outcome. Second, no a priori sample size or power calculation was performed, and the small sample produced imprecise adjusted estimates with wide confidence intervals. Third, multiple secondary and exploratory comparisons were not adjusted for multiplicity. Moreover, six patients contributed more than one restoration; intra-patient clustering was not modelled in the main analysis, and the one-restoration-per-patient sensitivity analysis does not fully resolve this issue. Fourth, the 6-month follow-up was insufficient to assess survival, secondary caries, debonding, wear or long-term failure.

Future studies should use prospective controlled designs with larger samples, a priori sample-size planning, and longer follow-up. Baseline clinical ratings should be incorporated into the primary analysis, and patient-level clustered models should be used when more than one restoration is included from the same participant. Quantitative assessment of marginal adaptation, wear, surface roughness and colour stability would strengthen outcome evaluation. Patient-centred outcomes should also be included.

## Conclusions

Both conventional indirect composite and CAD/CAM hybrid composite onlay workflows showed clinically acceptable short-term outcomes in this single-centre retrospective cohort. CAD/CAM hybrid composite onlays showed more favourable unadjusted 6-month clinical ratings, but there was no statistically significant adjusted association for the principal outcome, marginal fit. Baseline imbalance, residual confounding, workflow-related differences, small sample size, unmodelled intra-patient clustering and short follow-up preclude conclusions of superiority. These preliminary findings require confirmation in adequately powered prospective studies with longer follow-up and baseline-adjusted, patient-clustered analyses.

## Figures and Tables

**Table 1:** Table 1: Baseline characteristics of the two groups (n=33 per group).

**Variable**	**Group 1 Conventional indirect composite**	**Group 2 CAD/CAM hybrid composite**	**SMD**
Age, mean ± SD (years)	21.5 ± 5.1	21.8 ± 5.3	0.058
Female, n (%)	26 (78.8)	26 (78.8)	0.000
Mandibular tooth, n (%)	24 (72.7)	23 (69.7)	0.066
Molar tooth, n (%)	22 (66.7)	21 (63.6)	0.055
Black Class II compound, n (%)	32 (97.0)	32 (97.0)	0.000
≥2 cusps lost, n (%)	26 (78.8)	25 (75.8)	0.072
Endodontically treated, n (%)	26 (78.8)	26 (78.8)	0.000

SMD: Standardised mean difference. An SMD below 0.1 indicates adequate balance between groups.

**Table 2:** Table 2: Proportions of Alpha ratings by group and time point (n=33 per group).

**Outcome / Time point**	**Group 1 n (%)**	**Group 2 n (%)**	*p* ** value**
Marginal fit - Baseline	25 (75.8)	32 (97.0)	0.028*
Marginal fit - 1 month	22 (66.7)	31 (93.9)	0.005*
Marginal fit - 3 months	20 (60.6)	29 (87.9)	0.011*
Marginal fit - 6 months (principal)	19 (57.6)	28 (84.8)	0.015*
Occlusal fit - Baseline	20 (60.6)	30 (90.9)	0.004*
Proximal contact - Baseline	25 (75.8)	30 (90.9)	0.105
Proximal contact - 1 month	23 (69.7)	29 (87.9)	0.072
Proximal contact - 3 months	22 (66.7)	28 (84.8)	0.089
Proximal contact - 6 months	19 (57.6)	26 (81.3)	0.037*
Anatomic form - Baseline	29 (87.9)	32 (97.0)	0.166
Anatomic form - 1 month	27 (81.8)	30 (90.9)	0.286
Anatomic form - 3 months	24 (72.7)	29 (87.9)	0.118
Anatomic form - 6 months	22 (66.7)	26 (78.8)	0.263
Colour/gloss - Baseline	26 (78.8)	31 (93.9)	0.144
Colour/gloss - 1 month	24 (72.7)	28 (84.8)	0.232
Colour/gloss - 3 months	24 (72.7)	26 (78.8)	0.563
Colour/gloss - 6 months	13 (39.4)	25 (75.8)	0.003*
Marginal discolouration - 1 month	29 (87.9)	33 (100)	0.039*
Marginal discolouration - 3 months	29 (87.9)	31 (93.9)	0.390
Marginal discolouration - 6 months	24 (72.7)	29 (87.9)	0.118
Restoration integrity - 1 month	33 (100)	33 (100)	-
Restoration integrity - 3 months	32 (97.0)	32 (97.0)	1.000
Restoration integrity - 6 months	28 (84.8)	32 (97.0)	0.195
Secondary caries absent - 6 months	32 (97.0)	33 (100)	0.315

*p<0.05 (chi-square test, or Fisher's exact test when expected frequency <5). No Charlie ratings were observed at any time point. Group 1, conventional indirect composite onlays; Group 2, CAD/CAM hybrid composite onlays.

**Table 3:** Table 3: Within-group temporal trends in Alpha ratings (Cochran’s Q test).

**Outcome**	**Group**	**Baseline % Alpha**	**1 mo % Alpha**	**3 mo % Alpha**	**6 mo % Alpha**	*p*
Colour/gloss	1	78.8	72.7	72.7	39.4	0.001*
	2	93.9	84.8	78.8	75.8	0.105
Anatomic form	1	87.9	81.8	72.7	66.7	0.024*
	2	97.0	90.9	87.9	78.8	0.046*
Marginal discolouration	1	-	87.9	87.9	72.7	0.049*
	2	-	100	93.9	87.9	0.050
Marginal fit	1	75.8	66.7	60.6	57.6	0.041*
	2	97.0	93.9	87.9	84.8	0.112
Proximal contact	1	75.8	69.7	66.7	57.6	0.047*
	2	90.9	87.9	84.8	81.3	0.392
Restoration integrity	1	-	100	97.0	84.8	0.030*
	2	-	100	97.0	97.0	0.368

*p<0.05 indicates a statistically significant within-group decline over time. Exploratory analyses not adjusted for multiple comparisons. Group 1, conventional indirect composite onlays; Group 2, CAD/CAM hybrid composite onlays. A dash (-) indicates that the criterion was not assessed at that time point.

## Data Availability

The datasets used and/or analysed during the current study are available from the corresponding author on reasonable request.
